# Deforestation Impacts on Bat Functional Diversity in Tropical Landscapes

**DOI:** 10.1371/journal.pone.0166765

**Published:** 2016-12-07

**Authors:** Rodrigo García-Morales, Claudia E. Moreno, Ernesto I. Badano, Iriana Zuria, Jorge Galindo-González, Alberto E. Rojas-Martínez, Eva S. Ávila-Gómez

**Affiliations:** 1 Centro de Investigaciones Biológicas, Instituto de Ciencias Básicas e Ingeniería Universidad Autónoma del Estado de Hidalgo, Mineral de la Reforma, Hidalgo, México; 2 División de Ciencias Ambientales, Instituto Potosino de Investigación Científica y Tecnológica A.C., San Luis Potosí, San Luis Potosí, Mexico; 3 Instituto de Biotecnología y Ecología Aplicada (INBIOTECA), Universidad Veracruzana, Xalapa, Veracruz, Mexico; University of Western Ontario, CANADA

## Abstract

Functional diversity is the variability in the functional roles carried out by species within ecosystems. Changes in the environment can affect this component of biodiversity and can, in turn, affect different processes, including some ecosystem services. This study aimed to determine the effect of forest loss on species richness, abundance and functional diversity of Neotropical bats. To this end, we identified six landscapes with increasing loss of forest cover in the Huasteca region of the state of Hidalgo, Mexico. We captured bats in each landscape using mist nets, and calculated functional diversity indices (functional richness and functional evenness) along with species richness and abundance. We analyzed these measures in terms of percent forest cover. We captured 906 bats (Phyllostomidae and Mormoopidae), including 10 genera and 12 species. Species richness, abundance and functional richness per night are positively related with forest cover. Generalized linear models show that species richness, abundance and functional richness per night are significantly related with forest cover, while seasonality had an effect on abundance and functional richness. Neither forest cover nor season had a significant effect on functional evenness. All these findings were consistent across three spatial scales (1, 3 and 5 km radius around sampling sites). The decrease in species, abundance and functional richness of bats with forest loss may have implications for the ecological processes they carry out such as seed dispersal, pollination and insect predation, among others.

## Introduction

Species richness is one of the most widely used measures of biodiversity [1–2]. It is a property of ecological communities that can be used to diagnose the impact of habitat modification and to suggest priorities for conservation. However, this measure assumes that all species contribute to ecosystem functioning in a similar way, thus providing an incomplete and limited perspective of the complexity of biodiversity [3]. Therefore, to understand the changes in biodiversity under different scenarios of human impact (e.g., habitat loss and fragmentation, or changes in land use), other aspects that are complementary to biodiversity should be evaluated, such as phylogenetic diversity or functional diversity [2, 4].

Functional diversity has received growing attention in studies of community ecology and conservation biology, because it may give us a better understanding on ecosystem functioning, resistance and resilience [5–6]. Functional diversity is the variability in functional roles played by species within ecosystems and represents the degree of functional differences among species, in for example, the way they use resources [7–8]. Functional diversity can be quantified using different indices that capture different aspects of the distribution of functional traits within a community, e.g., [9–10]. Functional traits are phenotypic, observable or operational characteristics that affect species performance or ecosystem processes [8]. An approach based on functional traits allows us to understand how different species and communities respond to habitat modification [1, 11].

In Latin America, 2.5 million hectares of tropical forests are cut annually for crop cultivation and animal husbandry [12]. In Mexico, deforestation has been greatest in the tropical regions, drastically reducing and degrading tropical forests [13]. Despite this loss of natural habitats, the Neotropics is still the most diverse region in the world in terms of vertebrates [14]. Neotropical bats are an abundant and very diverse group that represent up to 50% of the local mammal species [15]. Additionally, they are easy to sample, their natural history and taxonomy are relatively well known and they have a variety of diets, foraging strategies, body sizes, flight capacities, and degree of specialization for different habitats [16]. Owing to these characteristics, they have been used to evaluate the effects of habitat changes [17]. Most of the studies that have addressed this topic however, have only focused on classifying bat species into functional groups based on their feeding guild or other behavioral characteristics [17–22]. A recent study has explored the relationships between bat species richness and their evolutionary histories, and how do phylogeny determine bat functional diversity in ecological communities [22]. But only recently, some studies have incorporated indices that include variability in functional traits [23]. Here we study bat functional diversity using quantitative methods with multiple functional traits, separating functional richness and functional evenness.

To evaluate the response of bat assemblages to habitat loss, it is necessary to measure the effects that occur within the area of movement of the different species each night [24]. Although very little is known about the movement patterns of Neotropical bats, we know that species can respond differently to anthropogenic disturbance owing to differences in their vagility, food and roost requirements, and their life history attributes [16, 25]. Moreover, in our study area we found that bats exhibit different thresholds to habitat loss depending on the spatial scale [26]. Some bats are more abundant in cluttered canopy sites, even though the landscape had been deforested; other species might be locally abundant in habitats with little canopy, but need landscapes that have not been deforested; and a third group of species require tree cover at all spatial scales [26]. Thus, to evaluate the effect of forest loss on the taxonomic and functional diversity of bats, here we follow a multiscale approach.

In this paper we test the hypothesis that bat species richness, abundance, and functional diversity are related to the loss of arboreal vegetation. We predict that as deforestation increases on the landscape, the richness, abundance and functional diversity of bats will decrease. Also, given that movement patterns and thresholds to habitat loss among species are scale-sensitive, we expect that the pattern of bat functional diversity loss with increasing deforestation will depend on the spatial scale. We focused in The Huasteca region in northeastern Mexico, which is an area of great biological interest because it is home to a large diversity of plants and animals that are of notable ecological and evolutionary value, and it is the largest expanse of evergreen rainforest remnants in the northern edge of the Neotropics [27].

## Methods

### Study area

The study was conducted in the state of Hidalgo, Mexico. The region is comprised of hills and mountain ranges spanning elevations of 18 to 200 m a.s.l. The climate is warm or semi-warm humid, with a mean annual temperature of 24°C and annual precipitation is 1200 to 3000 mm. The rainy season is from June to October and the dry season is from November to May [28]. The main native vegetation is semi-deciduous tropical forest, with secondary vegetation in different stages of succession [29]. Recently, conservation and climate change mitigation programs have been set in motion at the regional level [30]. However, vast areas of the native vegetation have been cut and burned over the last three decades, in favor of agriculture [29]. Currently, cattle ranching and crop cultivation have resulted in the disappearance of 50% of the natural vegetation cover [29]. More details and a map of the study area can be found in [26].

### Site selection and plant cover classification

Six sampling sites were selected, separated by a minimum of 10 km to ensure their spatial independence [26]. While these sites cover a representative gradient of deforestation, neither sites with 100% of their original forest cover intact, nor those that have been totally transformed into anthropogenic habitats were included because they are not present in the region.

We described each study site based on its landscape composition, using the supervised classification technique, with satellite images for 2007 and 2008 from Google Earth at a resolution of 1 m^2^ per pixel, which were the most recent at the time of the study. We identified three land cover types: forest cover, farming lands and urban areas. The first category includes native vegetation (semi-deciduous tropical forest), riparian corridors and disturbed vegetation with arboreal cover, i.e. the remnants of tropical forest and young or mature secondary vegetation, with a canopy height of 5 to 15 m. Some representative arboreal species are *Brosimum alicastrum*, *Bursera simaruba*, *Cedrela odorata*, *Protium copal*, *Cecropia obtusifolia* and *Muntigia calabura*, with the shrub layer characterized by *Acacia cornigera*, *Bauhinia americana*, *Bocconia frutescens*, *Piper* sp. and *Solanum* sp. [28, 31]. Farming lands include agricultural fields sown after the partial or total removal of the native vegetation, and pastures. The main crops in the region are corn (*Zea mays*), beans (*Phaseolus vulgaris* and *P*. *coccineus*), guava (*Psidium guajava*), citrus fruit (*Citrus sinensis* and *Citrus limon*), and sugar cane (*Saccharum officinarum*). Pastures for cattle are included in this category, and they are characterized by cultivated grass species such as *Paspalum* sp. and *Andropogon* sp. These pastures also have isolated *Ficus* trees that are left standing by the ranchers to provide shade for cattle. *Rubus* sp., *Kalanchoe pinnata* and *Bidens pilosa* have also colonized these areas [29]. The urban areas include towns and settlements with houses, industrial construction and other types of permanent infrastructure.

We delimited three concentric circles (landscape units) around each sampling site, each with a different radius: 1, 3 and 5 km. The largest (5 km) and smallest (1 km) scales were selected as they encompass the area over which the largest and smallest species of bats (*Artibeus* sp. and *Glossophaga soricina*, respectively) respond to habitat loss [32]. Other studies have used the same methodology of circular landscape units with 1, 3 and 5 km radius to evaluate the influence of habitat loss on bats [24, 26, 33–34]. In each landscape unit, we quantified the area occupied by each cover type using the program ArcView 3.2. To evaluate habitat loss, we used the percent forest cover relative to the total cover for each landscape unit (Table 1), so units represent a gradient of vegetation loss around each sampling site. Sampling site 1 had the highest forest cover percentage, while site 6 was the most deforested. Sites are evenly distributed along the forest cover gradient at a 1 km scale (Table 1). Unfortunately, at the 3 km scale we did not find landscapes with intermediate forest cover. We verified the areas for the different cover types by ground truthing (i.e., ground surveys to verify that all landscape features were the same as in the satellite images and that no important changes had occurred since the images were taken in 2007–2008).

**Table 1:** Percent of forest vegetation cover for the six sampling sites in the Huasteca region, in the state of Hidalgo, Mexico. For each site, vegetation cover was measured in three spatial scales (buffers with different radius around sampling sites).

	Landscape scale		
Sampling site	1 km	3 km	5 km
1	66.29	72.41	85.96
2	58.68	71.28	71.25
3	36.10	35.72	50.14
4	32.53	30.43	38.97
5	27.27	30.19	28.01
6	13.16	27.62	23.42

### Bat captures

At each site, we sampled bats for three nights spaced across the rainy season (July to October 2011, with at least one month between samples at the same site), as well as three nights during the dry season, with the same time lag separation (February to April 2012), for a total of six sampling nights per site. This sampling effort has been reported as the minimum to complete bat inventories in similar environmental conditions [35]. To get accurate estimators of bat diversity, we carried out this sampling effort at each site, instead of expanding our study to increase the number of sampling sites. On each sampling night, we captured bats in eight hanging mist nets (12 × 2.5 m) at the ground level. Every sampling night the nets were placed in the same locations within each study site. To increase the probability of capture, we hung the nets in places with a reasonable amount of arboreal vegetation and when possible, over trails that bats might use as flight paths. We left the nets open for six hours starting at dusk (1800–2400 h approximately), except when there was a full moon to avoid capture bias.

We identified bats to the species level using the field guide of [36], and taxonomic nomenclature was based on that of [37]. We limited the analysis to bats belonging to the families Phyllostomidae and Mormoopidae because they belong to a natural group (superfamily Noctilionoidea) whose distribution is limited exclusively to the Neotropics [38]. Also, given that these species can easily be caught with mist nets in the understory, potential bias is avoided when estimating diversity with this sampling method [35, 39]. Aerial insectivores (Emballonuridae, Molossidae and Vespertilionidae families) were excluded from the study because they can be underrepresented when only mist nets are used [39].

### Ethics Statement

Field sampling was done on public and private lands, with the corresponding permission of owners. We worked under the authority of scientific collecting permit SGPA/DGVS/05036/11 obtained from the Mexican Council for the Environment and Natural Resources (SEMARNAT, Secretaría del Medio Ambiente y Recursos Naturales), which legislates scientific field samplings in Mexico. We freed all bats where they had been caught, on the same night. We did not perform any other activities that required specific permissions. For field sampling in Mexico, the approval by an Institutional Animal Care and Use Committee (IACUC) or equivalent animal ethics committee is not required.

### Functional trait selection

We used four functional traits for each bat species: weight, size, diet and wing morphology (Table 2). We selected these traits under the assumption that they characterize the ecological segregation of the species in the community and give a broad overview of the importance of each bat species with respect to the different ecological processes they participate in [18, 40]. For example, body weight and size reflect the type and quantity of resources consumed by a species [41]. To quantify body weight, we weighed each bat and averaged the values to estimate mean body weight per species. For these measurements, we did not include pregnant females since their inclusion would introduce bias into the estimates of this functional trait. We measured bat size as mean forearm length per species (S1 Table).

**Table 2:** Functional traits used to determine the functional diversity of the bat community in the Huasteca region of the state of Hidalgo, Mexico. Functional groups of the frugivorous species (S1 Fig): F1: *Sturnira hondurensis* and *S*. *parvidens*, F2: *Artibeus lituratus* and *A*. *jamaicensis*, F3: *Carollia perspicillata* and *Dermanura tolteca*, and F4: *Chiroderma salvini*.

Type of datum	Functional trait	Attribute	Functional value
Categorical	Diet	Fruit	F1, F2, F3 and F4
		Nectar and pollen	Nectarivorous
		Blood	Hematophage
		Insects	Insectivorous
Numerical	Weight	Body weight	Mean for the species (g)
	Size	Forearm	Mean length for the species (cm)
	Wing morphology	Wing loading	Mean for the species
		Aspect ratio	Mean for the species

For diet, we obtained quantitative data for frugivorous species because there are variations in their main food items. For example, in the Huasteca region *Artibeus jamaicesis* use mainly *Ficus* fruits, while *Sturnira hondurensis* consumes more *Piper* fruits [42]. For bats with other feeding habits, diet was qualitatively evaluated, and species were assigned to the following trophic guilds based on the available literature for each of them: nectarivores (*Glossophaga soricina* and *Leptonycteris yerbabuenae*), insectivores (*Pteronotus davyi* and *P*. *parnellii*), and hematophages (*Desmodus rotundus*). To describe the diet of the frugivorous bats, fecal samples were collected from a plastic sheet (12×1 m) placed below each mist net [43]. The fecal samples were analyzed in the laboratory to search for seeds. The seeds were washed, examined under a dissecting microscope and identified to the lowest possible taxonomic level (S2 Table). To avoid over-representing the diet of frugivorous bats as a functional trait in the analysis, categorical groups were created as a function of their diet. To this end, a similarity dendrogram based on Morisita’s index was generated (S1 Fig) using the cluster method with a single linkage algorithm in the program PAST [44].

Wing morphology was included as a functional trait, as it provides a general view of the ecology and behavior of species [40, 45–47]. We measured wing loading and aspect ratio as wing descriptors whose ecomorphological relationships have been previously described [45]. In general terms, species with short, wide wings have low wing loading and aspect ratio values, indicating that they fly slowly but are agile and can easily maneuver through vegetation, thus preferring forested habitats. In contrast, bats with long, narrow wings have high wing loading and aspect ratio values, indicating that they fly at high speeds but have little maneuverability, preferring open habitats with few obstacles [45]. To quantify these two descriptors, we took photographs of the right wing of at least 15 bats per species. We analyzed the photos using ImageJ 1.45s (National Institutes of Health, U.S.A.), to measure the length of the forearm (ab), the wingspan (E), and the total area of the wing (A). Finally, body weight (W) was calculated as mean mass multiplied by 9.81 ms^-2^, acceleration due to gravity. With these data, wing loading was calculated as: WL = W/A; and the aspect ratio as: AR = E^2^/A [45].

### Data analysis

To analyze species inventory completeness by sampling site we calculated the Abundance-based Coverage Estimator (ACE), a nonparametric species richness estimator based on the species’ abundances [48], with the program EstimateS version 8.0 [49]. We did this analysis separately for each season (rainy and dry), and also using the data from both seasons together. Inventory completeness was estimated as the percent represented by the species richness recorded in the field relative to the maximum richness expected according to ACE.

We quantified bat species richness, abundance, and two indices of functional diversity for each site. Bat richness was measured as the number of species, and abundance as the number of individuals caught. The functional richness index (FD) measures the total length of the branches of a functional dendrogram generated using a cluster analysis [10, 50]. The functional evenness index (FEve) [51] measures the relative representation of the functional traits within the community [52] and quantifies the regularity with which species abundance is distributed in a multidimensional functional space [51]. Gower’s coefficient was used as a measure of the ecological distance between species since this measure is the most suitable one when using quantitative and qualitative values for functional traits [53]. However, to guarantee their equality in the analysis, the functional traits of the species were standardized using the following function: *Z*_*i*_ = (*x*_*i*_-*x* mean)/SD, where *Z*_*i*_ is the normalized value of the trait for species *i*, *x*_*i*_ is the trait value for species *i*, *x* mean is the mean value of a trait for all species and SD is the standard deviation in trait values across all bat species. The functional diversity indices were calculated using the software FDiversity [53]. We calculated species richness, abundance, functional richness, and functional evenness for each sampling night, and also cumulative values of these community parameters for each study site during the rainy and the dry seasons.

To test the hypothesis that as deforestation increases bat diversity will decrease, we performed generalized linear models with community parameters (richness, abundance, FD, and FEve) as response variables. We used Poisson error distributions for species richness, negative binomial for abundance, binomial distribution for FEve, and Gaussian distribution for FD (given that this was the only normally distributed variable, according to the Shapiro-Wilk W test, P>0.12). In all cases, the fitted model was Y = μ + Forest cover + Season + ε. We also tested for the interaction between forest cover and season, but it was not significant in any case, and models improved when we removed this interaction term (lower deviance and Akaike information criterion). To explore how the relationships change with the spatial scale of deforestation, the models were repeated with forest cover at 1, 3 and 5 km scales. We did these models in the R 3.0 software (R Development Core Team, 2013, available from: https://www.r-project.org).

## Results

We captured 906 bats belonging to the Phyllostomidae and Mormoopidae families, representing 10 genera and 12 species (Table 3). The most abundant species were *Sturnira hondurensis* and *Artibeus jamaicensis*, with 224 and 218 bats each, respectively. During the dry season *S*. *hondurensis* was the most abundant species, while *A*. *jamaicensis* was the most abundant during the rainy season. With the sampling effort made, inventory completeness was greater than 90% for the majority of the sampling sites (Table 4). Values lower than this were only recorded for two sites: site one for both seasons together (76.98%) and during the rainy season (87.03%), and site four during the dry season (86.08%). In general terms, these results indicate that a notable portion of the species present at the different sites was recorded.

**Table 3:** List of bat species captured. For each species we include the total number of individuals recorded in each site of the Huasteca region in the state of Hidalgo, Mexico.

Family	Species	Site 1	Site 2	Site 3	Site 4	Site 5	Site 6
Mormoopidae	*Pteronotus davyi*	1	0	0	0	0	0
	*Pteronotus parnellii*	1	2	0	0	0	0
Phyllostomidae	*Artibeus jamaicensis*	106	24	26	35	14	13
	*Artibeus lituratus*	67	21	7	21	13	20
	*Carollia perspicillata*	0	4	0	0	0	0
	*Chiroderma salvini*	5	3	0	0	2	1
	*Dermanura tolteca*	0	2	0	0	0	0
	*Desmodus rotundus*	12	2	5	12	20	0
	*Glossophaga soricina*	9	7	3	5	9	21
	*Leptonycteris yerbabuenae*	1	1	0	7	0	5
	*Sturnira hondurensis*	83	42	25	26	36	12
	*Sturnira parvidens*	55	22	40	24	25	8

**Table 4:** Cumulative parameters of bat communities. Data correspond to six sampling sites in the Huasteca region, Hidalgo, Mexico.

Site	Abundance	Species richness	Estimated richness (ACE)	Inventory completeness (%)	Functional richness (FD)	Functional evenness (FEve)
**Both seasons**						
1	340	10	12.99	76.98	4.46	0.39
2	131	11	11	100	4.82	0.43
3	106	6	6	100	2.64	0.68
4	130	7	7	100	2.96	0.68
5	119	7	7	100	3.12	0.77
6	80	7	7	100	2.95	0.62
**Dry season**						
1	95	7	7	100	3.12	0.73
2	59	8	8	100	3.32	0.73
3	38	6	6	100	2.64	0.73
4	36	6	6.97	86.00	2.64	0.71
5	35	7	7	100	3.12	0.76
6	22	5	5	100	2.15	0.78
**Rainy season**						
1	245	10	11.49	87.00	4.46	0.42
2	72	10	10.33	96.80	4.59	0.50
3	68	5	5	100	2.15	0.57
4	94	7	7	100	2.96	0.71
5	84	7	7	100	3.12	0.76
6	58	7	7	100	2.95	0.62

### Functional traits

For the diet of the frugivorous species, 305 fecal samples containing seeds were collected and these contained 17 morphospecies of which seven were identified to the species level and eight to the level of genus, with only two that could not be identified (S2 Table). The most frequent plant families in the diet of these bats were Solanaceae (103 samples) and Moraceae (73 samples). The cluster analysis returned four functional groups of frugivorous bats: the first with *Sturnira parvidens* and *S*. *hondurensis*, the second with *A*. *lituratus* and *A*. *jamaicensis*, the third with *Carollia perspicillata* and *Dermanura tolteca*, and the fourth with only *Chiroderma salvini* (S1 Fig).

We took morphological measurements from 105 bats. The majority of the bat species caught had a low aspect ratio (broad wings) and high wing loading (S1 Table). These characteristics allow these bats to make fast and short flights through the vegetation [45]. Although our analysis of functional richness (FD) relies on the total length of the branches in a functional dendrogram, and not in the number of functional groups, using the cluster analysis we can identify eight functional groups in our study area (S3 Table).

### Bat responses to deforestation

**Fig 1:**
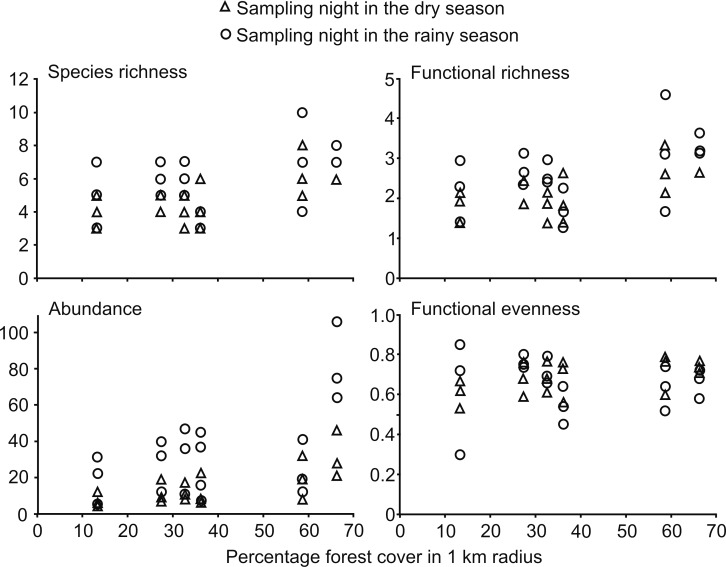
Bat community parameters per sampling night in relation to forest cover in the Huasteca region, Hidalgo, Mexico. As an example, forest cover is shown when it is measured in the buffers of 1 km radius around sampling sites, given that all the results are consistent at the 3 measured spatial scales. Six sampling nights were carried out at each one of the six sites (total n = 36).

Cumulative values of bat diversity parameters per sampling site were lower during the dry season than in the rainy season (Table 4). Species richness, abundance and functional richness per night are positively related with forest cover (Fig 1). Generalized linear models show that there is a significant effect of forest cover in these three parameters, while seasonality had a clear effect on abundance, and a marginal effect on functional richness. All these findings were consistent at the three spatial scales (Table 5).

**Table 5:** Summary of generalized linear models results. Bat diversity parameters (species richness, abundance, functional richness and functional abundance) were tested as dependent variables. In all cases, the fitted model was Y = μ + Forest cover + Season + ε.

	1 km				3 km				5 km			
	Coefficient estimate	S.E.	z / t value	P	Coefficient estimate	S.E.	z / t value	P	Coefficient estimate	S.E.	z / t value	P
**Species richness**												
Intercept	1.04	0.29	3.62	<0.01***	1.00	0.29	3.43	<0.01***	1.06	0.29	3.69	<0.01***
Forest cover	0.01	<0.01	2.19	0.03*	0.01	<0.01	2.35	0.02*	0.01	<0.01	2.02	0.04*
Season	0.19	0.14	1.30	0.19	0.19	0.14	1.30	0.19	0.19	0.14	1.30	0.19
Null deviance, d.f.			18.09, 35				18.09, 35				18.09, 35	
Residual deviance, d.f.			11.65, 33				11.01, 33				12.37, 33	
AIC			143.58				142.94				144.30	
**Abundance**												
Intercept	0.97	0.37	2.64	<0.01**	0.99	0.39	2.56	0.01*	0.99	0.37	2.66	<0.01**
Forest cover	0.02	<0.01	4.46	<0.01***	0.02	<0.01	3.99	<0.01***	0.02	<0.01	4.30	<0.01***
Season	0.80	0.19	4.28	<0.01***	0.80	0.19	4.12	<0.01***	0.80	0.19	4.24	<0.01***
Null deviance, d.f.			75.57, 35				69.43, 35				74.81, 35	
Residual deviance, d.f.			36.40, 33				36.58, 33				36.57, 33	
AIC			281.14				284.37				281.68	
**Functional richness**												
Intercept	0.97	0.39	2.47	0.02*	0.85	0.39	2.18	0.04*	1.02	0.41	2.50	0.02*
Forest cover	0.02	0.01	3.45	<0.01**	0.02	0.01	3.84	<0.01***	0.01	<0.01	3.05	<0.01**
Season	0.43	0.21	2.06	0.05*	0.43	0.20	2.13	0.04*	0.43	0.21	2.00	0.05
Null deviance, d.f.			18.77, 35				18.77, 35				18.77, 35	
Residual deviance, d.f.			12.60, 33				11.84, 33				13.37, 33	
AIC			72.37				70.14				74.50	
**Functional evenness**												
Intercept	0.75	1.35	0.55	0.57	0.79	1.39	0.57	0.57	0.82	1.37	0.60	0.55
Forest cover	<0.01	0.01	0.19	0.84	<0.01	0.02	0.13	0.89	<0.01	0.01	0.11	0.91
Season	-0.12	0.70	-0.18	0.85	-0.13	0.71	-0.18	0.86	-0.13	0.71	-0.18	0.86
Null deviance, d.f.			1.95, 35				1.95, 35				1.95, 35	
Residual deviance, d.f.			1.87, 33				1.89, 33				1.90, 33	
AIC			37.21				37.29				37.32	

Significance codes

* P<0.05

** P<0.01

*** P<0.001.

## Discussion

This study documents the decrease in the species and functional richness of bat species along a gradient of habitat loss in Mexico using functional traits. The consequences of habitat loss on Neotropical bats were previously studied with respect to changes in bat richness and abundance among different habitats e.g., [17, 20], among different successional stages of vegetation e.g., [54–55], at the landscape scale e.g., [24, 33–34], and analyzing trophic guilds as a way of evaluating the functional diversity of the group e.g., [18–21, 56]. In our study, functional diversity was evaluated using multivariate methods based on the functional traits of this group of bats, an approach that allows us to assess the impact of forest loss. According to our hypothesis, bat functional richness decreases as deforestation increases.

Although our sampling effort was enough in most of the studied sites, the lack of inventory completeness at site one may be related to the large forest cover that might harbor other bat species such as *Carollia sowelli*, *Diphylla ecaudata* and *Micronycteris microtis*, which have been reported for other sites in the Huasteca region [57]. During the dry season at site four, however, we recorded only six species, two of them with a single bat (*Desmodus rotundus* and *Glossophaga soricina*). It is likely that by increasing the sample, more of these individuals would have been caught since they are not rare in the region. More sampling effort would also had benefitted this work by allowing us to increase the number of sampling sites with intermediate deforestation, given that landscape units with more than 70% of forest cover are no longer present in the region. Moreover, as explained, our study is restricted to families Phyllostomidae and Mormoopidae, which are easily caught with mist nets in the understory and display a variety of diet preferences. It still remains unexplored the potential impact of aerial insectivores (Emballonuridae, Molossidae and Vespertilionidae families) on the functional diversity of bat communities, but this must be done with different sampling methods such as acoustic bat detectors or harp traps, and with a detailed analysis of the insect groups that make up bat diet.

Our results do not support the initial idea regarding the dependence of the relationship between deforestation and bats on the spatial scale, given that the effects of deforestation on species and functional richness were consistent at three spatial scales. This suggests that differences in home range and in thresholds to habitat loss among species do not reflect on community parameters.

### Neotropical bat richness and abundance

Bat richness increased with increasing forest cover area following the species-area pattern, one of the most robust empirical generalizations in ecology [58]. There are several underlying mechanisms for this species-area relationship, such as habitat heterogeneity and susceptibility to extinction, which may interact simultaneously to determine this pattern [58]. For bats, it has been demonstrated that the size of an area is important in determining (or at least predicting) the species richness of a given place [59]. In different Neotropical sites there is greater bat diversity in areas with ample forest cover e.g., [16, 32–34, 54]. However, other studies have suggested that bat diversity may be higher in patchy landscapes with a mosaic of different habitat types and relatively small forest remnants e.g., [60]. Therefore, sites with high percentage of continuous forest, as well as patchy landscape mosaics, may be important for maintaining bat species diversity in the Huasteca region.

The specific response of each bat species to habitat loss depends on their characteristics, such as body size, wing morphology, foraging and echolocation strategies, which determine their presence and abundance under certain conditions [61–62]. For example, small insectivorous mormoopid bats disappear from deforested areas because they are more successful feeding where vegetation is dense than in open areas [63]. Accordingly, we only captured four individuals of the genus *Pteronotus* in sites with the highest percentage of forest cover. Also, some species such as *Carollia perspicillata* uses hollow trunks and tree leaves as refuge [64], so the absence of suitable refuges in deforested sites could limit their presence. These species are generally present in continuous habitats, but can also use riparian vegetation and corridors that cross deforested areas, though they will not leave the protection offered by the vegetation to venture into open areas [25]. Thus, small species such as *Carollia* sp., *Dermanura* sp. and *Glossophaga soricina* could be limited to areas with a high degree of plant cover because, unlike larger species (e.g., *Artibeus* sp.), they cannot fly great distances to move across the whole deforested landscape [65]. For *Desmodus rotundus*, a recent analysis that included not only landscape but local canopy cover as explanatory variables [26], showed that this species might be locally abundant in habitats with little canopy, but needs landscapes that have not been deforested.

Bat abundance was affect both by forest cover and seasonality because bats were abundant only during the rainy season at the site with greatest forest cover (up to 106 individuals per night). This is due to the high abundance of *Artibeus* and *Sturnira* species, and may be related with food availability, such as the presence of fructifying plants. In the other 33 nights we captured fewer than 50 individuals, regardless of forest cover. Thus, bat abundance may be related to seasonal variations in food availability for bats in the Huasteca region [42].

Our data reveal a relationship between the functional diversity of bat communities and landscape composition, i.e. the landscape area that remains covered by forest. For phyllostomid bat assemblages it has been noted that landscape composition has a stronger impact than landscape spatial configuration [66], however the influence of landscape configuration (e.g., degree of landscape fragmentation, forest edge density) on bat functional diversity still needs to be tested. Some small understory bats can use resources from the matrix, particularly from secondary forests, agroforestry crop systems such as coffee plantations, and riparian corridors. Thus, both landscape composition and configuration are important for bats, because different patches provide different and sometimes complementary resources (e.g. fruiting trees, refuge and roosting sites).

### Functional diversity of Neotropical bats

Functional richness (FD) was clearly related to the degree of forest cover measured around sampling sites, but functional evenness (FEve) was not. Similar results have been reported for bat communities in Costa Rica [23]. This suggests that the response of functional diversity to habitat loss depends on the functional aspect evaluated. FD may be related to species richness [51, 60], and in our study the sites with greater forest cover had greater bats species richness. The absence of insectivorous species (*P*. *davyi* and *P*. *parnellii*), and some frugivorous (*C*. *perspicillata* and *D*. *tolteca*) led to the low functional richness values obtained in the deforested sites. These species have extreme values for some functional traits, their unique functional characteristics indicate that they might be key species in the region. For example, among the species studied, *P*. *davyi* and *P*. *parnellii* have the lowest wing loading values. *C*. *perspicillata* has the lowest aspect ratio value and *D*. *tolteca* has the highest wing loading value (S1 Table). This means that these species have extreme wing morphology values. StrauB et al. [67] suggest that if species richness changes, but functional diversity remains constant, additional species or those that disappear do not exhibit unique ecological traits and can be considered functionally redundant. The low abundance in our study area of the insectivorous species and two frugivorous species, and their disappearance from our two least forested sites could foretell the loss of important functional traits from deforested areas, but this needs further exploration. Bat species traits are correlated with landscape attributes (composition and configuration). For example, the number foraging strategies and foraging locations are negatively correlated with forest cover and mean patch size, while wing morphology traits are positively correlated with an increase in pastures in man-modified landscapes [23]. The disappearance of functional traits from could have negative implications for the control of insect populations and the reproductive success of the plants these bats feed on, especially those that are abundant at the study sites (e.g., *Ficus* sp., *Solanum* sp., *Cecropia* sp., *Physalis* sp.). Although these plants are not exclusively pollinated and/or dispersed by bats, seed production and seedling recruitment could decrease in sites where bat functional diversity is low.

In our study area, functional richness was only marginally affected by seasonality. But in Costa Rica landscape effects on bat functional diversity are season specific [23]: areas with intermediate amounts of forest and pasture during the dry season harbored highest bat functional diversity, while during the wet season this occurred in areas with large, compact forest patches. However, contrary to our measure of functional richness, these measures of functional diversity (Rao’s quadratic entropy) include bat abundance [23]. As we have explained, bat abundance is also highly affected by seasonality in our study area, probably due to resources availability. Considering that functional evenness takes abundance into account, the communities with a similar number of individuals (evenness) among functional groups (i.e. groups of species that are similar in their functional traits), and regular distances between functional groups (similar lengths of the segments of the minimum spanning tree) have higher functional evenness values. That is, if a species disappears, but another that belongs to the same functional group is present, this second species can compensate in abundance for the species that is absent, maintaining functional evenness even though species richness is lower compared with that of other communities. The opposite occurs when a few species dominate the community and all belong to the same functional group, resulting in the overrepresentation of functional traits, or when some functional groups are underrepresented, functional evenness decreases.

Shleuter et al. [68] suggests that a high FEve value indicates that the habitat might not be very structurally complex, with few interactions between species, few available niches, and that the niches might be occupied by species evenly. The structure of the vegetation is one of the axes along which species are distributed, allowing for niche differentiation and coexistence within the community [69]. Structurally complex habitats can have a wider variety of microclimates, more resources and life forms that exploit those resources, and more microhabitats for refuges and roosting sites [70], allowing them to maintain a greater number of species. Although we did not quantify vegetation complexity, it is possible that sites with less forest cover have also less complexity and niches, thus leading to high functional evenness.

### Implications for conservation

To date biodiversity conservation efforts have focused almost exclusively on the number of species, even though several authors have highlighted the importance of avoiding strategies that only make use of species richness for making decisions regarding conservation [4, 14, 71]. The measurement of the functional traits of species in this study allowed us to identify, in addition to the decrease in species richness and abundance, the decrease in the functional richness of the bat community and the increase in functional evenness.

In this paper we found that bat species and functional richness are related to landscape composition, particularly to the loss of forest. These results suggest that mosaic landscapes with forest remnants are of important conservation value for bat species and the ecosystem services they provide. For example, frugivorous bats are important seed dispersal agents that favor the regeneration of the vegetation [42]. Nectarivorous and insectivorous species also provide ecological services of great value such as pollination and insect pest population control [72].

Given that the contributions of the different species to ecosystem function differ so much, conservation efforts should incorporate an evaluation of functional diversity, complementary to that of species richness. By quantifying functional diversity in natural communities, researchers gain additional understanding about the importance of species to ecosystem processes and this can affect how areas are prioritized for conservation [7, 71] and the relationship among bat taxonomic, functional and phylogenetic diversity in tropical environments [22]. Therefore, the use of functional richness as a tool for the conservation of the bat community in the Huasteca region could guarantee that the environmental services that they participate in continue to be provided.

## Supporting Information

S1 Fig Dendrogram of similarity used to form functional groups based on the diet of the frugivorous species of bats. (PDF)

S1 Table Feeding guild and wing morphology descriptors of the bats from six landscapes in the Huasteca region, Hidalgo, Mexico. (PDF)

S2 Table Seeds collected from the droppings of seven frugivorous bats in Huasteca region of the state of Hidalgo, Mexico. (PDF)

S3 Table Bat functional groups identified with the functional dendrogram used to calculate functional richness (FD), and functional traits of the species. (PDF)
