# Supplementary material for: Deforestation Impacts on Bat Functional Diversity in Tropical Landscapes
Source: PLoS One. 2016 Dec 7;11(12):e0166765. doi: 10.1371/journal.pone.0166765 (PMC5142789; doi:10.1371/journal.pone.0166765)

Deforestation Impacts on Bat Functional Diversity in Tropical Landscapes

Rodrigo García-Morales, Claudia E. Moreno, Ernesto I. Badano, Iriana Zuria, Jorge Galindo-González, Alberto E. Rojas-Martínez & Eva S. Ávila-Gómez

S1 Fig. Dendrogram of similarity used to form functional groups based on the diet of the frugivorous species of bats. The analysis is based on data from S2 Table. Ajam: *Artibeus jamaicensis*, Alit: *A. lituratus*, Cper: *Carollia perspicillata*, Csal: *Chiroderma salvini*, Dtol: *Dermanura tolteca*, Shon: *Sturnira hondurensis*, Spar: *S. parvidens*.

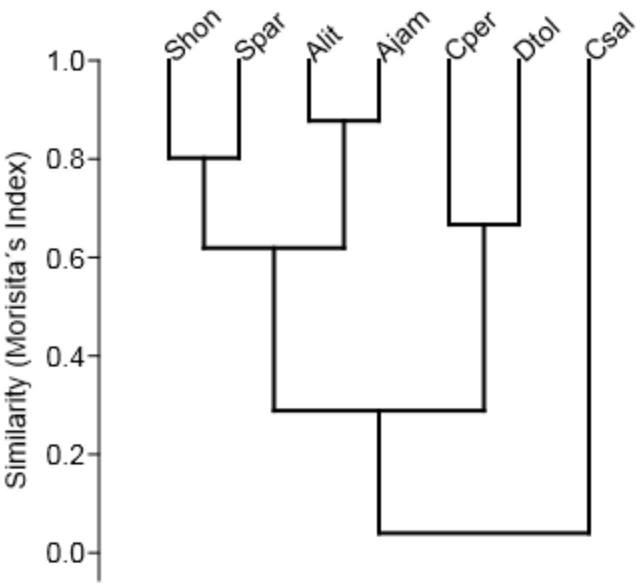

Supplement: S1 Fig — (PDF) [file pone.0166765.s001.pdf]
