# Supplementary material for: Deforestation Impacts on Bat Functional Diversity in Tropical Landscapes
Source: PLoS One. 2016 Dec 7;11(12):e0166765. doi: 10.1371/journal.pone.0166765 (PMC5142789; doi:10.1371/journal.pone.0166765)
Supplement: S1 Table — (PDF) [file pone.0166765.s002.pdf]

Deforestation Impacts on Bat Functional Diversity in Tropical Landscapes

Rodrigo García-Morales, Claudia E. Moreno, Ernesto I. Badano, Iriana Zuria,
Jorge Galindo-González, Alberto E. Rojas-Martínez & Eva S. Ávila-Gómez

S1 Table. Feeding guild and wing morphology descriptors of the bats from six landscapes in the Huasteca region, Hidalgo, Mexico. For each wing morphology descriptor we present mean ± standard deviation. *n*= number of individuals, ab= forearm length, W= body weight, E= wingspan, A= total wing area. AR= aspect ratio, WL= wing loading. *= measures included in the analyses of functional diversity.

	Guild	n	ab*	W*	E	A	AR*	WL*
Artibeus jamaicensis	Frugivorous 2	15	5.97±0.18	41.00±8.41	37.17±1.93	121.46±11.98	11.47±1.20	3.21±0.62
Artibeus lituratus	Frugivorous 2	10	6.83±0.12	64.00±6.08	43.16±1.68	166.75±7.47	11.19±0.80	3.78±0.46
Carollia perspicillata	Frugivorous 3	4	4.03±0.04	20.25±1.09	27.85±1.02	75.61±2.22	10.29±0.85	2.63±0.20
Chiroderma salvini	Frugivorous 4	11	4.61±0.09	27.91±4.27	34.60±0.84	84.64±2.69	14.17±0.81	3.25±0.55
Dermanura tolteca	Frugivorous 3	2	3.70±0.06	21.50±5.50	25.39±0.23	52.39±0.80	12.31±0.42	4.04±1.09
Desmodus rotundus	Hematophage	10	5.57±0.26	37.60±10.60	36.38±1.16	92.18±5.05	14.44±1.40	4.02±1.16
Glossophaga soricina	Nectarivorous	12	3.60±0.10	11.50±1.80	24.28±1.46	45.88±3.14	12.96±1.74	2.47±0.44
Leptonycteris yerbabuenae	Nectarivorous	7	5.10±0.45	25.57±3.33	32.47±6.31	65.22±12.01	17.42±5.77	3.92±0.52
Sturnira hondurensis	Frugivorous 1	15	3.97±0.12	19.53±2.47	26.83±1.78	62.32±4.05	11.61±1.44	3.07±0.26
Sturnira parvidens	Frugivorous 1	15	3.73±0.25	17.33±2.70	28.09±1.79	42.77±4.83	11.35±1.53	4.03±0.77
Pteronotus davyi	Insectivorous	1	4.50	7.00	26.80	47.30	15.18	1.45
Pteronotus parnellii	Insectivorous	3	5.93±0.09	20.00±0.82	34.53±1.80	88.07±2.42	13.55±1.09	2.23±0.09
