# Supplementary material for: Deforestation Impacts on Bat Functional Diversity in Tropical Landscapes
Source: PLoS One. 2016 Dec 7;11(12):e0166765. doi: 10.1371/journal.pone.0166765 (PMC5142789; doi:10.1371/journal.pone.0166765)
Supplement: S2 Table — (PDF) [file pone.0166765.s003.pdf]

Deforestation Impacts on Bat Functional Diversity in Tropical Landscapes

Rodrigo García-Morales, Claudia E. Moreno, Ernesto I. Badano, Iriana Zuria, Jorge Galindo-González, Alberto E. Rojas-Martínez & Eva S. Ávila-Gómez

S2 Table. Seeds collected from the droppings of seven frugivorous bats in Huasteca region of the state of Hidalgo, Mexico. For each plant we report the number of bat fecal samples that included seeds.

Family	Species	Ajam	Alit	Cper	Csal	Dtol	Shon	Spar
Cecropiaceae	*Cecropia obtusifolia*	0	1	0	0	0	0	2
	Coussapoa purpusii	6	6	0	0	0	23	10
Moraceae	*Ficus* sp.1	6	2	0	0	0	11	8
	Ficus sp.2	1	0	0	0	0	2	0
	Ficus sp.3	0	2	0	0	0	3	1
	Ficus sp.4	0	3	0	0	0	5	1
	Ficus sp.5	9	10	0	0	0	0	2
	Ficus sp.6	3	2	0	1	0	1	0
Piperaceae	*Piper amalago*	2	1	0	0	0	1	1
	Piper hispidum	2	2	1	0	1	7	16
Solanaceae	*Markea* sp.	1	1	0	0	0	4	4
	Physalis sp.	8	4	1	0	0	20	46
	Solanum diphyllum	1	0	0	0	0	1	5
	Solanum rudepanum	1	0	0	0	0	1	5
Ulmaceae	*Trema micrantha*	1	0	0	0	0	6	14
Unknown	Unknown1	2	1	0	0	0	10	10
	Unknown 2	1	0	0	0	0	1	1

Ajam: *Artibeus jamaicensis*, Alit: *A. lituratus*, Cper: *Carollia perspicillata*, Csal: *Chiroderma salvini*, Dtol: *Dermanura tolteca*, Shon: *Sturnira hondurensis*, Spar: *S. parvidens*.
