# Supplementary material for: Deforestation Impacts on Bat Functional Diversity in Tropical Landscapes
Source: PLoS One. 2016 Dec 7;11(12):e0166765. doi: 10.1371/journal.pone.0166765 (PMC5142789; doi:10.1371/journal.pone.0166765)
Supplement: S3 Table — (PDF) [file pone.0166765.s004.pdf]

Deforestation Impacts on Bat Functional Diversity in Tropical Landscapes

Rodrigo García-Morales, Claudia E. Moreno, Ernesto I. Badano, Iriana Zuria,
Jorge Galindo-González, Alberto E. Rojas-Martínez & Eva S. Ávila-Gómez

S3 Table. Bat functional groups identified with the functional dendrogram used to calculate functional richness (FD), and functional traits of the species. For this analysis, the matrix with the values of species functional traits of each site is converted into a distance matrix used to generate a dendrogram, and the distances represented by each branch of the dendrogram are added for each site (Petchey and Gaston 2002b). The relative abundance of the species is not taken into account (Petchey and Gaston 2006).

Functional group	Species	Diet	Body size	Weight (g)	Aspect ratio	Wind loading
1	*Pteronotus davyi*	Insectivorous	4.50	7	15.2	1.5
2	*Pteronotus parnellii*	Insectivorous	5.93	20	1.09	0.1
3	*Glossophaga soricina*, *Leptonycteris yerbabuenae*	Nectarivorous	3.60- 5.10	21.03-25.42	12.95-17.42	2.47-3.92
4	*Sturnira hondurensis*, *S. parvidens,*	Frugivorous 1	3.73-3.97	19.63-20.45	11.6-18.56	3.06-4.02
5	*Dermanura tolteca*, *Carollia perspicillata*	Frugivorous 3	3.70- 4.03	20.25-21.50	10.28-12.31	2.63-4.04
6	*Desmodus rotundus*	Hematophage	5.57	37.84	14.43	4.01
7	*Artibeus jamaicensis*, *A. lituratus*	Frugivorous 2	5.97- 6.83	55.62-65.52	11.19-11.46	3.20-3.78
8	*Chiroderma salvini*	Frugivorous 4	4.61	27.9	14.16	3.24
